# Chinese version of narcolepsy severity scale: a validation study

**DOI:** 10.1186/s12883-019-1570-5

**Published:** 2019-12-21

**Authors:** Hui Ouyang, Fang Han, Qiwen Zheng, Jun Zhang

**Affiliations:** 1 Department of Neuromedicine, Peking University People’s Hospital, 11 Xizhimen South Street, Beijing, China; 2 Department of Pulmonary Medicine, Peking University People’s Hospital, Beijing, China; 3 Department of Epidemiology and Biostatistics, School of Public Health, Peking University Health Science Center, Beijing, China

**Keywords:** Symptom evaluation, Reliability, Validity, Psychometric, Questionnaire

## Abstract

**Background:**

The narcolepsy severity scale (NSS) was developed to measure the severity and consequences of symptoms in patients with narcolepsy. The scale has been validated in France, though no other studies have further validated this instrument. The current study aimed to present psychometric properties and describe the score distribution of the Chinese-NSS.

**Methods:**

One hundred twenty-two patients with narcolepsy (41 females and 81 males; mean age 26.14 ± 15.40 years) participated in the study. All patients completed the Chinese-NSS. Cronbach’s α, item-total score correlations, exploratory factor analysis (EFA), and correlations between NSS total scores and clinical or sleep parameters were then calculated.

**Results:**

EFA yielded a three-factor model. Internal consistency was acceptable (Cronbach’sα = 0.799). The NSS total score had significant correlations with the Epworth sleepiness score (0.447), pediatric daytime sleepiness scale (0.318), the insomnia severity index (0.592), Beck depression inventory (0.593), EurQol five dimensions-utility (0.457), EurQol five dimensions -VAS (− 0.323), the sleep disturbance scale for children (0.440), the children depression inventory (0.553), and the pediatric quality of life inventory (0.555) total scores, demonstrating acceptable convergence as predicted.

**Conclusions:**

The current study is the first validation study of the narcolepsy severity scale in an Asian population. The findings validated the Chinese-narcolepsy severity scale in a Chinese population with acceptable psychometric properties. There are minor differences between our results and those of the original study due to cultural differences.

## Background

Narcolepsy is a life-long neurological disorder with onset primarily during childhood and adolescence [1, 2]. Worldwide, the prevalence of narcolepsy ranges from 0.002 to 0.167%. The main symptoms of narcolepsy are excessive daytime sleepiness (EDS), cataplexy, sleep paralysis, hallucinations, and disrupted nighttime sleep [3–5]. Patients with narcolepsy also suffer from other symptoms including mood disorders and behavioral and attention disorders [6] .

Most subjective tests that are currently used to evaluate the severity of symptoms [7–9], such as the Epworth sleepiness score (ESS) and the pediatric daytime sleepiness scale (PDSS) mainly focus on EDS. Other symptoms of narcolepsy are often not properly monitored. To overcome this limitation, Dauvilliers [9] et al. developed the Narcolepsy Severity Scale (NSS) in 2017 to measure the severity of the five main symptoms of narcolepsy. This scale, which consists of 15 items, primarily assesses the frequency and severity of excessive daytime sleepiness, cataplexy, hypnagogic hallucinations, sleep paralysis, and disrupted nighttime sleep. The NSS shows good psychometric properties (internal consistency, convergent validity, and structural validity) and has been shown to be able to detect changes in French narcolepsy patients’ symptoms following treatment [9]. As such, the NSS has the potential to be a valid and reliable screening instrument for assessing the symptoms of narcolepsy patients.

However, no other studies have further validated this instrument in clinical and research settings. It has also not been translated into Chinese and is somewhat difficult to use with Chinese young patients because in China, people who are younger than 18 are not allowed to drive. Thus, the item “To what extent these sudden daytime sleep episodes affect your ability of driving?” is not suitable for China’s social environment. A process of cross-cultural adaptation and validation was necessary to further validate this instrument and ensure that it is suitable for use in China. Thus, the current study aimed to: 1) translate and culturally adapt the NSS to the Chinese language; 2) test the preliminary validity and reliability of a version of the “Narcolepsy severity scale” (NSS) that has been translated into Chinese and adapted to evaluate the severity and consequences of symptoms in Chinese narcolepsy patients; 3) identify cut-off values to distinguish between treated and non-treated patients. This scale, which is focused on the severity of narcolepsy symptoms, is expected to contribute to the assessment of narcolepsy symptoms and changes in symptoms after treatment in Chinese narcolepsy patients.

## Methods

### Ethical considerations

This study was approved by the local ethics committee and conducted in accordance with the guidelines of the Institutional Review Board of Peking University People’s Hospital, China. Written informed consent was obtained from all participants.

### Participants

For this study, 122 consecutive patients with narcolepsy (41 females and 81 males; mean age 26.14 ± 15.40 years) were randomly selected from the sleep laboratory of the Peking University People’s Hospital, a unit that evaluates patients with sleep disorders and receives referrals from all over China. Thus, the selected sample can be considered to adequately represent the Chinese narcolepsy population. The inclusion criteria were patients who were diagnosed with narcolepsy following the ICSD-3 criteria [10] and who were willing to participate in interviews. Patients were excluded from the study if they could not fill out the questionnaire (for example, patients who were younger than 8 or have serious psychiatric disorders). A total of 5–10 subjects were recommended for each item to achieve the validity and reliability studies [11].

### The study process

This study was conducted in three phases. In phase 1, the NSS was translated from English to Chinese. Phase 2 involved pre-testing the pre-final version of the translated questionnaire. A validation study was carried out in phase 3. This study spanned from June 2018 to May 2019.

Phase 1 (Translation and adaptation process).

The original English questionnaire [9] was translated following the translation, back translation, and cross-cultural adaptation processes recommended by Beaton et al. [12] and Guillemin et al. [13] to ensure that the contents and meanings were preserved. The forward translation was carried out by two translators, including a certified translator (linguist) and a clinician (for the medical terms), both of whom are bilingual, bicultural, native Chinese speakers. The translators worked independently to produce a Chinese version of the questionnaire that was later back-translated to English. The translators worked independently from each other. An expert panel composed of the translators and the researchers met to compare the versions that had been translated into Chinese. The best translations were merged, after which a cultural adaptation of several of the terms that had given rise to discussion was carried out until a consensus was reached. At the end of this meeting, a final harmonized Chinese version of the NSS was obtained. Item 7 of the questionnaire was adapted to “To what extent these sudden daytime sleep episodes affect your ability of driving a car (for students: classroom performance)?” to better fit the Chinese social environment.

Phase 2 (Pre-test procedure).

Ten narcolepsy patients took the pre-test for the Chinese version of the NSS in order to discuss the pertinence of the items and to identify comprehension problems for narcolepsy patients. These patients were selected randomly from the target population. Face validity was confirmed and wording and contents were checked in the pilot survey. Each patient was asked whether the words and terms used in the Chinese version were clear, relevant, and comprehensible. In this way, patients identified their difficulties and discussed them afterwards. Both adult patients and pediatric patients agreed that the NSS was straightforward and easy to understand. After the face validity measure, the evaluation committee composed of three translators and five neurologists met again and formulated the final Chinese version of the questionnaire (Chinese-NSS), considering several points that had led to questions from patients. This version had the same number of items and domains as the original version, with several minor adjustments. Finally, the experts evaluated the content validity (items and domains) of the Chinese version.

The Chinese-NSS is composed of 15 items and is divided into 3 subscales. The first subscale concerns sleepiness, the second subscale concerns cataplexy, and the third subscale concerns hallucination, sleep paralysis, and disturbed nighttime sleep.

Phase 3 (Fig. 1).

**Fig. 1:**
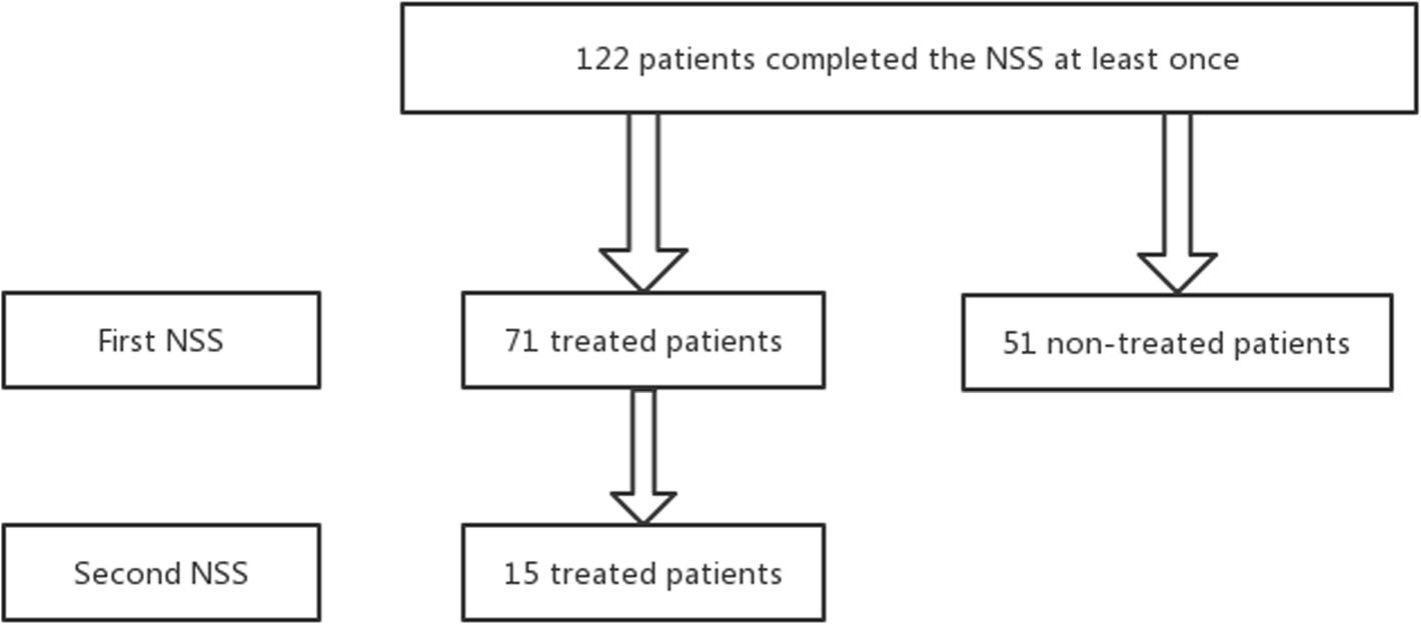
Study flow chart

The NSS was later tested for its reliability and validity among 122 subjects in the same institute who were selected from the patients referred to the sleep laboratory of the Peking University People’s Hospital. To be included in the study, all patients were required to be able to write, read, and speak Chinese fluently. Pediatric participants’ parents helped them complete the questionnaire to ensure that it was filled in properly. This study was conducted in a single center and the study participants were patients diagnosed with narcolepsy. According to the recommendations of Everitt [14], we opted for a sample size ranging from 100 to 200. Patients were asked to fill in the patient information sheet as well as the NSS, which took them about 20–30 min to complete. Total scale scores for the NSS, as well as the Epworth sleepiness score ESS [7], the insomnia severity index (ISI) [15], the Beck depression inventory (BDI) [16], the EurQol five dimensions (EQ-5D) [17], the PDSS [18], the sleep disturbance scale for children (SDSC) [19], the children depression inventory (CDI) [20], and the pediatric quality of life inventory (PedsQL) [21] were calculated according to standard scoring procedures.

### Statistical methods

Statistical analyses were carried out using SPSS version 22.0. Test-retest reliability was conducted and internal consistency was assessed using the Cronbach’s alpha coefficient. Demographic characteristics and clinical data were described using means and standard deviations for continuous variables and percentages or frequencies for categorical variables. The independent Student’s t-test was used to compare continuous variables between independent groups, and the Chi-square or Fisher’s exact tests were used for categorical variables. Associations between continuous variables were assessed using the Spearman correlation coefficient. To analyze NSS factor structure, a principal components factor analysis was conducted using a varimax rotation. Sampling adequacy was measured by calculating the Kaiser-Meyer-Olkin (KMO) index. Statistical significance was set at *p* < 0.05.

## Results

### Demographic and descriptive statistics

In total, 122 consecutive patients with narcolepsy were included in this study (53 pediatric patients and 69 adult patients), all of whom were Chinese. The mean age of the narcolepsy patients was 26.14 ± 15.40 years; 33.6% were female (*n* = 41) and 66.4% were male (*n* = 81). Among them, 78 were consecutive untreated and 44 were consecutive treated. Among the treated patients, 13 completed the NSS scale a second time after a median interval of 60 days (range: 14–128 days). According to the tests of normality (K-S test), the distribution of the total scores for the narcolepsy population was in line with normal distribution (*p* = 0.079). There were no differences in NSS scores between the total scores of the pediatric group and the adult group (26.12 + 8.04 and 28.41 + 12.25, *p* > 0.05).

### Internal consistency

The Cronbach’s α value was 0.799 for the whole sample and ranged from 0.697–0.817 for the subscales (factors) measuring excessive daytime sleepiness (EDS), cataplexy, sleep paralysis, and hallucination. Correlations between individual items and the total score of the NSS were significant and positive in the whole sample. The correlations of individual items with the total score ranged from 0.242–0.677.

These results indicated an acceptable level of internal consistency (Table 1 and Table 2).

**Table 1:** Item-total score correlations and Cronbach’s Alpha of NSS ^a^

Dimensions and items	Item-total score correlation	
	Spearman correlation coefficient	significance
Item 1	0.361	p < 0.001
Item 2	0.440	p < 0.001
Item 3	0.630	p < 0.001
Item 4	0.532	p < 0.001
Item 5	0.242	*p* = 0.007
Item 6	0.400	p < 0.001
Item 7	0.342	p < 0.001
Item 8	0.603	p < 0.001
Item 9	0.655	p < 0.001
Item 10	0.616	p < 0.001
Item 11	0.611	p < 0.001
Item 12	0.677	p < 0.001
Item 13	0.542	p < 0.001
Item 14	0.632	p < 0.001
Item 15	0.455	p < 0.001
Cronbach’s α of the NSS	0.799	
Alfa Cronbach global split-half coeficients	0.657	0.774

^a^ *NSS* narcolepsy severity scale

**Table 2:** Narcolepsy severity scale items-Factor analysis

Items	Communlities	Factors		
		I	II	III
1	0.334		0.457	
2	0.520		0.711	
3	0.776		0.845	
4	0.515		0.573	
5	0.478		0.412	
6	0.368		0.600	
7	0.257		0.462	
Cronbach’ α	0.697			
8	0.727			0.837
9	0.691			0.794
10	0.563			0.629
15	0.296			0.379
Cronbach’ α	0.740			
11	0.630	0.775		
12	0.759	0.859		
13	0.596	0.767		
14	0.692	0.814		
Cronbach’ α	0.817			
KMOa measure of sampling adequacy	0.728			
Percentage of cumulative variance explained	0.55			

a*KMO* Kaiser-Meyer-Olkin

### Reproducibility

Fourteen of the treated patients completed the NSS scale a second time after a median interval of 60 days (range: 14–128 days) (unchanged drugs and dosages). The investigators and test environments were exactly the same as in the patients’ first completion of the questionnaire. All of the patients that participated in the test-retest described their symptoms as stable and unchanged between the first and second measurements. In terms of the test-retest reliability of the Chinese-NSS, the reliability coefficient (intra-class correlation coefficients (ICC)) was 0.923 (95% CI: 0.779–0.975, *p* < 0.001).

### Content validity

Face validity was confirmed and wording and contents were checked in the pilot study. The subjects agreed that the NSS was straightforward and easy to understand. After the face validity measure, the evaluation committee composed of the translators and neurologists met again and formulated the Chinese-NSS. Finally, the experts confirmed that the content validity of the Chinese version was good.

### Structural validity

In the whole sample, the Kaiser-Meyer-Olkin (KMO) index was 0.728, confirming the sampling appropriateness, with sufficient association between variables to perform factor analysis. Based on the scree plot and Kaiser’s eigenvalues-greater-than-one rule, the current study confirmed the validity of a three-factor model, with eigenvalues higher than 1 that explained 54.6% of the total variance. FactorIwas composed of 4 items on sleep paralysis and hallucinations (questions 11, 12, 13, and 14), factorII included 7 items on EDS (questions 1, 2, 3, 4, 5, 6, and 7), and factor III included 4 items on cataplexy and nighttime sleep (question 8, 9, 10, and 15). Communalities, which can be interpreted as the proportion of each variable’s variance that can be explained by factor analysis, were generally higher than 0.47 (0.478–0.776), except for items 1, 6, and 15. The item loading values ranged from 0.381–0.696.

### Convergent validity

The convergent validity was calculated by comparing the correlations between NSS total scores and clinical or sleep parameters using Spearman’s correlation coefficient. All of these instruments have been reported to have sufficient measurement properties in our target population. The NSS total score significantly and positively correlated with ESS (*p* < 0.001), ISI (*p* < 0.001), BDI (*p* < 0.001), and EQ-5D (*p* = 0.001) in adult patients and correlated with PDSS (*p* = 0.038), SDSC (*p* = 0.004), CDI (*p* < 0.001), and PedsQL (*p* < 0.001) in pediatric patients. Descriptive statistics as well as the correlations between the NSS and other clinical or sleep parameters are shown in Table 3.

**Table 3:** Association between narcolepsy severity scale total score and rating scale scores

	Adult patients (*N* = 69)		Pediatric patients (*N* = 53)	
	Narcolepsy severity scale		Narcolepsy severity scale	
Measurements	correlation coefficient	*p*-value	Correlation coefficient	p-value
ESS^a^	0.447	< 0.001		
BDI-II^b^	0.593	< 0.001		
ISI^c^	0.592	< 0.001		
EQ-5D-Utility^d^	0.457	< 0.001		
EQ-5D-VAS^e^	−0.323	0.018		
PDSS ^f^			0.318	0.036
CDI ^g^			0.553	< 0.001
SDSC^h^			0.440	0.004
PedsQL^i^			0.555	< 0.001

^a^*ESS* Epworth sleepiness score

^b^*BDI-II* Beck depression inventory-II

^c^*ISI* the insomnia severity index

^d^*EQ-5D-Utility* EurQol five dimensions-Utility

^e^*EQ-5D-VAS* EurQol five dimensions-visual analog scale

^f^*PDSS* pediatric daytime sleepiness scale

^g^*CDI* children depression inventory

^h^*SDSC* sleep disturbance scale for children

^i^*PedsQL* pediatric quality of life inventory

### Discriminative validity

The results of the study with 51 drug-free patients and 71 treated patients showed that the NSS total score was significantly higher in the untreated than treated group (30.08 + 9.14 versus 25.44 + 11.21, *p* = 0.017). The range of the scores of the drug-free group was 9 to 55, while the range of the scores of the treated group was 4 to 51. The descriptive statistics are shown in Table 4.

**Table 4:** The demographic and NSS^a^ total score of drug-free and treated narcolepsy patients

variables	Drug-free patients *N* = 51		Treated patients *N* = 71		
	n	%	n	%	p-value
Gender					
male	37	72.55	44	61.97	0.249
female	14	27.45	27	38.03	
age	29.04 + 16.08		24.05 + 14.66		0.100
NSS total score	30.08 + 9.14		25.44 + 11.21		0.017

^a^*NSS* narcolepsy severity scale

### Floor or ceiling effects

The distribution of the total scores for the narcolepsy population was near the center of the possible range of scores, without a ceiling effect. They ranged from 0 to 50. None of the patients reached the highest score (57), and only 0.8% of the patients reached the lowest score (0).

## Discussion

To the best of our knowledge, this is the first study to examine the psychometric properties of a version of the NSS adapted for use in China. In our study, the questionnaire was slightly modified to ensure its suitability for the Chinese cultural context. We conducted pre-test interviews to record patients’ facial expressions and any misunderstandings of the scale. Through this procedure, the content of the questionnaire was judged to be applicable to Chinese native speakers. In addition, the acceptability and comprehension of the questionnaire have been confirmed.

This research examined the validity and reliability of the Chinese-NSS in the measurement of the main symptoms of narcolepsy and their changes after treatment. The measurement properties indicate that the Chinese version of the NSS is an effective and reliable tool for assessing the severity of narcolepsy symptoms and detecting changes in clinical symptoms after treatment. Children and adolescent patients also participated in this study, and there were no differences in the NSS scores of the pediatric and adult group. Hence, the results show that the questionnaire can be applied to juvenile patients, to a certain degree.

The reliability of the scale was assessed by internal consistency using Cronbach’s alpha, item-total correlations, and test-retest. The high Cronbach’s alpha coefficient for the scale, the results of the test-retest, and the acceptable corrected item-total coefficients for all items confirmed that the Chinese version of the NSS has good internal consistency, with the correspondent items properly correlated. The item-total score correlation coefficients were acceptable, being generally higher than 0.30 except for item 5. The results for internal consistency were similar to those obtained in Dauvillier’s studies. The original version of the questionnaire reported a lower item-total score correlation for item 5, but the researchers chose to keep this item because of its clinical significance [9]. The high correlation coefficient shows that the items of the scale have a strong correlation with the scale construct. Internal consistency of each sub-dimension, which was represented by the Cronbach’s alpha coefficients, ranged from 0.697–0.817. High Cronbach’s alpha coefficients indicate that the Chinese-NSS is structurally consistent and balanced [22]. The Cronbach’s alpha value of the original scale was 0.799. Based on these results, the Cronbach’s alpha values obtained by our study and by Dauvilliers et al. were consistent [23]. The results of the test-retest showed that the stability of responses to items was good. The results of reproducibility were similar to the original French version of the NSS.

Exploratory factor analysis was conducted to identify potential sub-scales of the NSS. Through exploratory factor analysis of the Chinese-NSS, 15 items of the scale were divided into three factors. The results of the exploratory factor analysis proved that the Chinese-NSS has acceptable structural validity. The three identified factors clarified 54.6% of the total variance, which is similar to the results of the exploratory factor analysis of the original scale [9]. When the principal component analysis and varimax orthogonal rotation method were used to evaluate the factor loads of each item, the item load higher than 0.35. However, the results of our study differ slightly from those of the original version in terms of item classification. The item “Currently, how disturbed is your nighttime sleep?” was part of factor 3 in our research but was included in factor 2 in the original study. Based on the grouping of items based on clinical expert criteria, disturbed nighttime sleep was not associated with any of the three factors, so this item is relatively independent from other items. On the other hand, the severity of disturbed nighttime sleep can both be associated with that of EDS and cataplexy, since they are all related to sleep instability. Therefore, the results of the factor analysis of our study and the original study, in which item 15 only partially overlapped with the assigned dimensions composing the scale, were reasonable and acceptable.

In line with the validation of the original version, there is a certain degree of differentiation of the scale because the total NSS score was significantly higher in the untreated than treated group. However, the ability to discriminate between the two groups was limited. This may be because some patients do not take drugs due to the mildness of their symptoms, and longitudinal studies should be performed in the future to evaluate the responsiveness of patients before and after drug usage.

Ideally, criterion validity would be measured with a gold standard when it is available. However, adequate tools that can be used as a “gold standard” to assess narcolepsy severity are missing [24, 25]. The convergent validity of the NSS was tested by analyzing the correlation between the scores of the NSS and those of other scales, showing statistically significant relationships [7, 8, 26–28]. We found positive and significant correlations between the NSS and ESS, ISI, BDI, EQ-5D, PDSS, SDSC, CDI, and PedsQL. The higher the correlation, the more valid the measure is.

Although the psychometric characteristics of the Chinese-NSS were similar to those of the original version, our results indicated that differences existed between the two versions due to cultural differences. First, the psychometric characteristics of the cataplexy-subscale (item 8–10) were better than items focusing on other symptoms of narcolepsy (EDS, sleep paralysis, and hallucination). This may be because there are more young narcolepsy patients in China than in European countries [29], and they usually have a higher frequency of cataplexy. Second, item 7 of the questionnaire was changed to “To what extent these sudden daytime sleep episodes affect your ability of driving a car (for students: classroom performance)?” to fit the Chinese social environment, thereby expanding the scope of the measure’s application. The results of our study have demonstrated that slight adaptations and wording changes are unlikely to affect the global score. The psychometric properties of item 7 were generally similar to those of the original version, although there was a lower community in our study. These small differences may due to the Chinese social environment.

Some limitations of this study should be acknowledged. First, since narcolepsy is a rare disease and the collection of cases is very difficult, the sample size used in our study is limited. Hence, the scale should be ascertained in further clinical trials as well as longitudinal assessments. Second, the interval for test-retest reliability is relatively long. Although during this period, patients’ conditions are stable and medication is regular, an interval less than 2 weeks would be preferable. Third, only patients older than 8 years old were included in the study, so this questionnaire may not be applicable to very young children.

## Conclusions

This is the second validation study of the NSS in the world and the first validation study of the NSS in the Asian population. The Chinese version of the NSS evidenced suitable psychometric characteristics in terms of reliability and validity for Chinese narcolepsy patients. However, our results indicated that despite cultural differences, the NSS can be used in clinical evaluations and in monitoring symptoms and complications in narcolepsy patients worldwide.

The Chinese version of the NSS is available as supplementary material. Supplementary materials related to this article can be requested by emailing the author.

## Data Availability

All experimental data and questionnaires within the article are available from the corresponding author upon reasonable request.

## Abbreviations

AUC: Area under curve
BDI: Beck depression inventory
BIC: Bayesian Information Criterion
CDI: Children depression inventory;
CFA: Confirmatory factor analysis
CFI: Comparative Fit Index
EFA: Exploratory factor analysis
EQ-5D: EurQol five dimensions
ESS: Epworth sleepiness score
ISI: The insomnia severity index
NSS: Narcolepsy severity scale
PDSS: Pediatric daytime sleepiness scale
PedsQL: Pediatric quality of life inventory
RMR: Root mean square residual
RMSEA: Root-mean-square error of approximation
ROC: Receiver operating characteristic
SDSC: Sleep disturbance scale for children
